# Highly Important

**Published:** 1848-07

**Authors:** 


### HIGHLY IMPORTANT.

Something like the following was published some forty years ago. With some sli ht alterations, it suits the times so admirably, that we give place to it:

"This is to give notice that Monsieur Roquet has lately arrived from Paris, and sells wholesale or retail all sorts of legs, arms, eyes, noses or teeth, made in the genteelest manner, and as now worn by persons of rank in France. He repairs and beautifies in a surprising manner any old, decayed, or lost parts of human bodies, fills up the wrinkles or furrows of old age, as well as the marks of small pox, carious TEETH, &c., with a *new invented paste*; for the latter purpose it stands unrivalled, actually making decayed teeth superior for usefulness to those which are perfectly sound—and will also supply the place of lost teeth, providing there be a small portion of the root left for attachment. He beautifies all bad shapes, by a new method of making a *gentle* incision to the bone, and filing off the protuberant parts. He also cures effectually the most stinking breaths, by extracting all decayed teeth and stumps, and burning the gums to the jaw bone, without the least pain or confinement, and puts in their stead, on a new principle, an entire set, so philosophically and mathematically fitted to the jaws, that people of the first fashion may eat, drink, swear, talk scandal, quarrel, and show their teeth, without the least indecency, inconvenience, or hesitation whatever.

N. B. Monsieur R. clears out a bruised eye, and replaces a bright *P ris* brilliant black, blue, or squinty one, red hot, without pain or loss of blood.

References,

Tom Pepper, Esq.,
Baron Munchausen, M. D.

